# Relative Contribution of Haptic Technology to Assessment and Training in Implantology

**DOI:** 10.1155/2014/413951

**Published:** 2014-02-20

**Authors:** David Joseph, Jean-Philippe Jehl, Pablo Maureira, Cyril Perrenot, Neal Miller, Pierre Bravetti, Pascal Ambrosini, Nguyen Tran

**Affiliations:** ^1^School of Surgery Nancy-Lorraine, University of Lorraine, 54505 Vandoeuvre-les-Nancy, France; ^2^Department of Periodontology, Faculty of Dentistry, University of Lorraine, 54000 Nancy, France; ^3^UMR-S1116, University of Lorraine, 54000 Nancy, France; ^4^Institut Jean Lamour, UMR 7198, CNRS, Université de Lorraine, 54011 Nancy, France; ^5^Collegium Santé, University of Lorraine, 54000 Nancy, France

## Abstract

*Background*. The teaching of implant surgery, as in other medical disciplines, is currently undergoing a particular evolution. *Aim of the Study*. To assess the usefulness of haptic device, a simulator for learning and training to accomplish basic acts in implant surgery. *Materials and Methods*. A total of 60 people including 40 third-year dental students without knowledge in implantology (divided into 2 groups: 20 beginners and 20 experiencing a simulator training course) and 20 experienced practitioners (experience in implantology >15 implants) participated in this study. A basic exercise drill was proposed to the three groups to assess their gestural abilities. *Results*. The results of the group training with the simulator tended to be significantly close to those of the experienced operators. *Conclusion*. Haptic simulator brings a real benefit in training for implant surgery. Long-term benefit and more complex exercises should be evaluated.

## 1. Introduction

Conventional training of a surgeon involves the acquisition of a number of skills, a long process that requires considerable attention to ultimately acquire a satisfactory base of knowledge, to facilitate apprehension and comprehension of situations, formulate diagnoses, guide decision making, and strengthen manual and technical skills thus leading to improved therapeutic abilities [1–3]. As higher surgical performance and standardization of training have become normative and safety objectives, awareness coming from the United States of America [4] and recently from the European Union [5] has stressed the urgency for institutions to invest in new teaching strategies such as simulation [6] and accredited residency-training programs in order to reduce variability in training methodology, technical skill, and the trainee's confidence and competence at the time of graduation [7].

In dental implantology, techniques and technologies are constantly evolving, and manufacturers are marketing new products that progressively help to push back a little more the limits of implant restoration. Whilst the act of drilling may seem at first relatively simple, implantology involves a much more complex global prosthetic treatment plan which requires full integration of key areas such as 3D structural acquisition, system operations, prosthetic requirements, visual representation, and *in fine* expectation of functional and aesthetic restoration. However, there is still no clear consensus in the certification of implant practitioners as recalled by the first European Consensus Conference in 2008 in Prague [8] stigmatizing, amongst other recommendations, very diverse training programs throughout Europe both at undergraduate and postgraduate levels that might hamper proficiency required in formative development.

Based on the performance of a basic act in implant surgery, the drilling procedure, the objective of our prospective study was to determine the contribution of a haptic simulator for simulating implant surgery, as a valuable teaching tool for training of third-year students in dentistry. The expected application of this research is to provide an adjuvant environment for learning implantology and to improve its assessment and proficiency.

## 2. Materials and Methods

### 2.1. General Study Design

The research was conducted at the School of Surgery of Nancy-Lorraine, Lorraine University, France. A total of 60 practitioners, dentists (*n* = 20) or third-year dental students (*n* = 40), all recruited from the Faculty of Dentistry, University of Lorraine, were included in this study. This prospective randomized observational study was approved by the BIOSE, the doctoral review board of the Lorraine University.

After informed consent, 40 third-year dental students were enrolled. Exclusion criteria were any previous experience with drilling training and with implant surgery. Students were then randomized into two groups: the “Novice” group (*n* = 20, 10 women and 10 men, mean age = 22 years) and the “Simulation” group (*n* = 20, 10 women and 10 men, mean age = 22 years). Both groups were instructed conventionally about the drilling from a PowerPoint presentation except that students in the “Simulation” group received prior individual training on the Virteasy simulator. Results were compared to an “Experienced” group which included 20 dentists (11 women and 9 men, mean age = 39.3 years) with a minimum experience of 15 placed implants (range: 15 implants to more than 800). Before starting any exercises in this study, people of this group had also received the PowerPoint presentation of drilling instruction.

### 2.2. The Simulator and Exercises

To be able to compare simulator trained students with untrained students and experienced practitioners, we started with the training of the simulator group.

**Figure 1:**
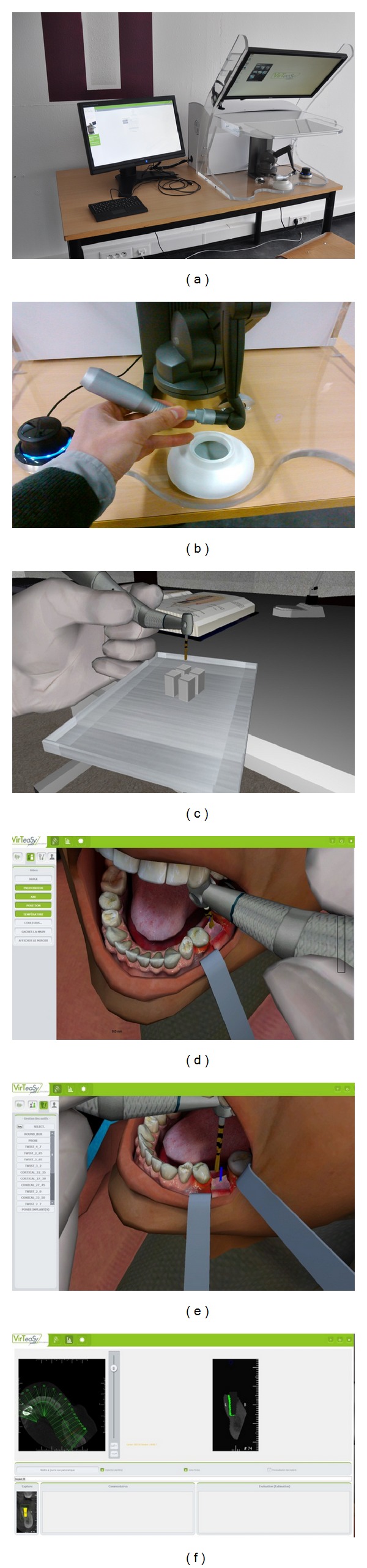
Haptic simulator and virtual exercises. (a) Overview of the simulator. (b) Positioning of the haptic contra-angled handpiece. (c) Image of the virtual drilling procedure. (d) Virtual implant site preparation. (e) Blue angulation guide. (f) Grading of virtual drilling (green is reference and black is actual drilling).

Virteasy is one of the first dental simulators on the market to reproduce the sensations of implant surgery. Briefly, it consists of a PC-type computer running Windows 7, a touch-screen control for interacting with simulator software, a 3D screen reflecting in a mirror, stereoscopic glasses for viewing the 3D scene, a plastic contra-angled handpiece connected to force feedback arm device (Phantom) to transcribe the tactile sensations of drill in the bone, and a foot pedal to start the virtual handpiece in the simulator (Figure 1(a)).

Before starting the exercise, participants performed simple exercises to familiarize themselves with the operation of the simulator, the use of virtual handpiece (Figure 1(b)), adjust their positions, and feel the feedback force provided by the machine. For example, a simple drill exercise on virtual blocks was proposed. These blocks mimicked the four bone densities described in the literature [9]; block 1 corresponds to a highly cortical bone and block 4 is a predominantly cancellous bone (Figure 1(c)). Using a virtual Astra 3.2 mm diameter drill to bore 11 mm deep, the exercise was repeated as many times as desired by the participant.

Then, each participant was given the same explanation of the implant selection and positioning, using specific scanner software. During this exercise, the students were familiarized with implant planning software and notions concerning diameter, implant shape, and positioning. Moreover, each participant was shown the mark on the drill not to exceed (11 mm).

After determining the location and the type of implant, each participant was asked to perform the procedure corresponding to the virtual “expert” planning furnished by the simulator. This reference is determined by the machine as the ideal planning. The intervention was carried out 8 times, 4 times in the presence of an instructor and 4 times in total autonomy. The simulator can provide assistance for the positioning of the point of impact and the three-dimensional positioning of the implant (Figure 1(d)). During the 8 trials, the student kept using the computer assistance to determine the point of impact which is the ideal position of the center of the future implant. Assistance to identify the three-dimensional position was provided only during the first 4 trials (Figure 1(e)).

The virtual material used for each test was a bur to mark the point of impact, a cylindrical 2 mm drill-driver, a cylindrical 2.85 mm drill, a 3.5 mm drill, and a conical 3.5/4.7 mm drill.

For each test, different parameters were recorded in an Excel spreadsheet:

- the position difference (in mm) relative to the reference position (position of the point of impact in relation to the position indicated by the simulator),
- the average-difference angle (°) with respect to the reference position (difference from the vestibular-lingual and mesial-distal angulation),
- the drilling depth (in mm, depth from the top of the ridge),
- the total time (in seconds) of the exercise,
- the actual drilling time (time in seconds during which the drill bit is rotated during the exercise),
- the eventual perforations identified visually (Figure 1(f)).

### 2.3. Procedure Presentation

Before drilling the resin model to evaluate their skills in implantology, all participants received basic instructions by means of a PowerPoint presentation explaining the different stages of implementation, the goals to achieve, and more particularly the pitfalls to avoid.

### 2.4. Creation of Synthetic Resin Model and Evaluation

To compare the three groups, we created a model stemmed from the scanner slices of the simulator exercise. The edentulous ridge was modeled using sheets of soft wax, 1 mm thick, reproducing the scanner slices from the simulator's software, at original scale. The different sheets were then assembled to form a block which was in turn inserted into a plaster model. An impression was made and a first plaster duplicate was made to verify the volume of the ridge and be sure it corresponded to the CT scan slices. Sixty resin (RenCast 52/53 Isocyanatethe FC/FC 52 Polyol) replicates were made from the master model. In order for the model to be radioopaque, 30% of barium sulphate was added. Several trials using liquid iodine were unsuccessful due to a problem of polymerization of the resin [10].

**Figure 2:**
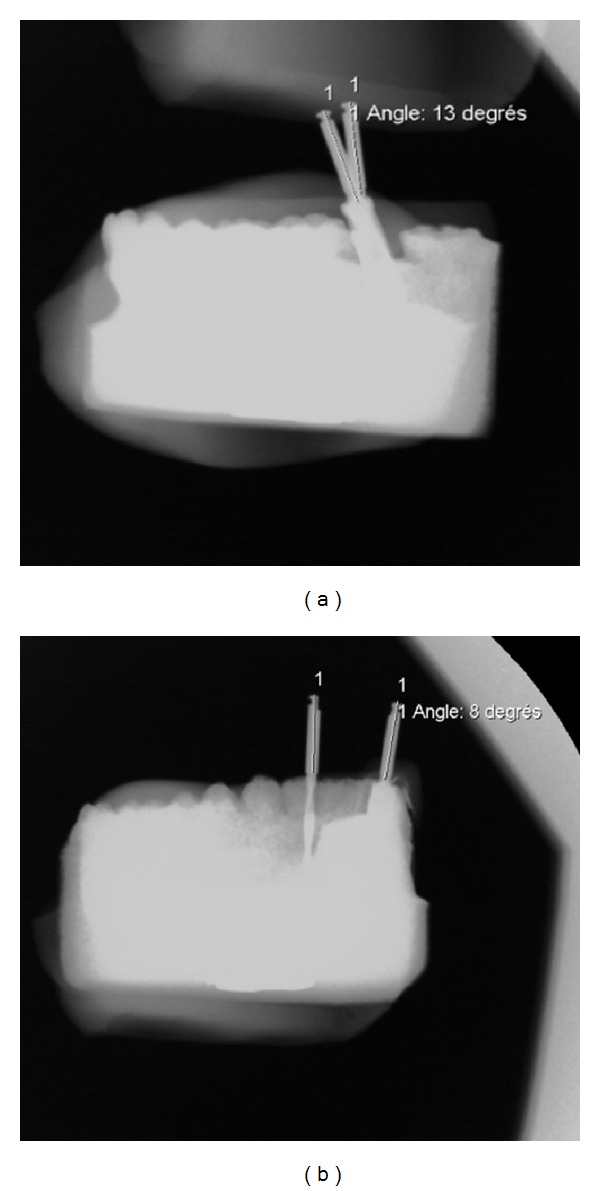
Evaluation of angle parameters with resin model. (a) Example of mesiodistal angle deviation. (b) Example of buccolingual angle deviation.

A transparent acrylic position key was used to calculate the angular deviation (mesiodistal and/or buccolingual) between the reference axis and the actual drilling of each model. To be sure that all measurements were performed in the same conditions, it was important that all models be scanned in the same way. For this purpose two silicone positioning bases were confectioned to stabilize the models in two well-defined positions, one to verify the mesiodistal angle and the other the buccolingual angle. The models were then passed through an X-ray image intensifier (ARCADIS Avantic, Siemens) and X-rayed in the two positions determined by the silicone bases to record the angle deviations (see Figure 2 for an example).

The measurements of the position difference were performed using a periodontal probe. An electronic caliper was used to determine the drilling depth by measuring the portion of the last drill emerging from the model.

All participants received a model with a random number of anonymity so that the evaluation would be blind. Using a graduated periodontal probe, participants were to mark the center of the ridge in the buccolingual and mesiodistal directions according to information provided in the presentation. Once the impact point is determined, the drilling of the implant was performed using a standard set of drills: a bur-haired and different cylindrical drill (2 mm, 3.2 mm, 3.7 mm, 4.2 mm, 4.7 mm, and 4.85 mm). Thus, the participants were confronted with various difficulties such as working positions and limitation of the buccal aperture of the manikin. The evaluation was focused on

- the correct centering of the targeted site,
- the presence or absence of perforation,
- the drilling depth,
- the deviation of the vestibulolingual direction relative to a reference axis on the control model,
- the deviation of the mesiodistal direction relative to a reference axis on the control model,
- the global drilling time.

### 2.5. Statistical Analysis

The results were expressed as mean ± standard deviation from the mean (m ± SEM). The one factor ANOVA or *t*-test two-tailed *t* was used to compare performance between groups. The frequency analyses were made using the Fischer test. Probability *P* < 0.05 was considered significant. Analyses were made possible through the GraphPad Prism (GraphPad Software San Diego, CA, USA).

## 3. Results

### 3.1. The Impact of Simulation Training on the Quality of Drilling

**Figure 3:**
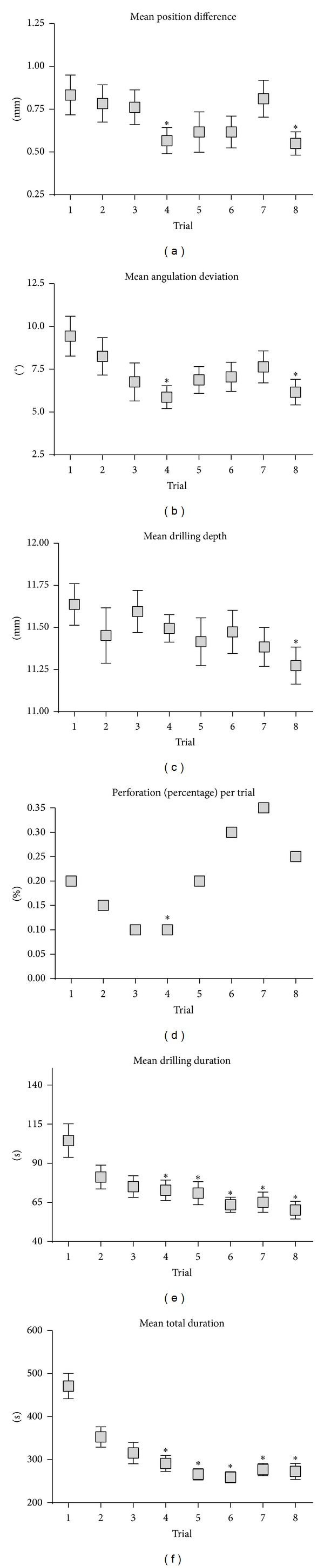
Evolution of drilling outcomes with simulator training. (a) Mean position difference. (b) Mean angulation deviation (mean of deviations for both buccolingual and mesiodistal angulations). (c) Mean drilling depth. (d) Perforation (percentage) per trial. (e) Mean drilling duration. (f) Mean total duration. Results are expressed as m ± SEM, *n* = 20. *P < 0.05 versus 1st trial.

Scores obtained on the simulator by the group of 20 participants from the third-year of dental surgery are displayed in Figure 3.

The progression of the centering precision is illustrated in Figure 3(a). A clear trial-dependent improvement was documented. The mean initial position was off the mark by 0.86 ± 0.12 mm, but after 8 exercises the deviation was reduced to 0.54 ± 0.06 mm (*t* = 2.247, *P* = 0.0310 versus the 1st trial). However, two phases of evolution could be identified. During the first 4 trials under guidance (position and angle), a progressive and significant improvement was documented; the mean position deviation for the fourth test was 0.55 ± 0.06 mm from the ideal center (*t* = 2.038, *P* = 0.04 versus the first trial). However this progression stagnated once the drilling angulation guidance was disabled.

Similar profile was documented with the difference in vestibulolingual and mesiolingual angulation (Figure 3(b)). Indeed, the difference in angulation was of 9.44 ± 1.16° initially and was gradually reduced to a value of 5.9 ± 0.67° after the fourth attempt (*t* = 2.66, *P* = 0.0121). When angulation virtual pointer was no longer available, accuracy stopped progressing. However, performance after the 8th trial was still significantly more accurate than after the initial trial, the angle deviation being 6.17 ± 0.94° (*t* = 2.297, *P* = 0.0288).

The drilling depth was also recorded and its change over time is shown in Figure 3(c). Unlike the two previous parameters, steady progress has been well highlighted here going from an initial drilling depth average of 11.64 ± 0.12 mm to an average of 11.27 ± 0.11 mm at the end of the training (*t* = 2,195, *P* = 0.0310).

For each trial, the presence or absence of perforations was determined (Figure 3(d)). Of the total 160 virtual trials, there were 33 cortical perforations made by different 13 participants, 11 while virtual guidance was activated and 22 when it was not. When in use, the virtual guidance greatly contributes to avoiding these occurrences.

Finally, the simulator measures speed in two different ways, the overall time and the actual drilling time (Figures 3(e) and 3(f)). Repeated practice with the simulator significantly improved these parameters. For instance, the initial drilling time was of 106 ± 46 sec and the initial overall total of 470 ± 131 sec. At the end of the training with simulator, all these parameters were significantly improved decreasing to 62 ± 26 sec for the drilling time and 272 ± 82 sec for the entire exercise (all *P* < 0.05 versus baseline).

### 3.2. Comparative Study of Drilling Parameters on the Outcome of Resin Model Scanners (Validity of Construction)

**Figure 4:**
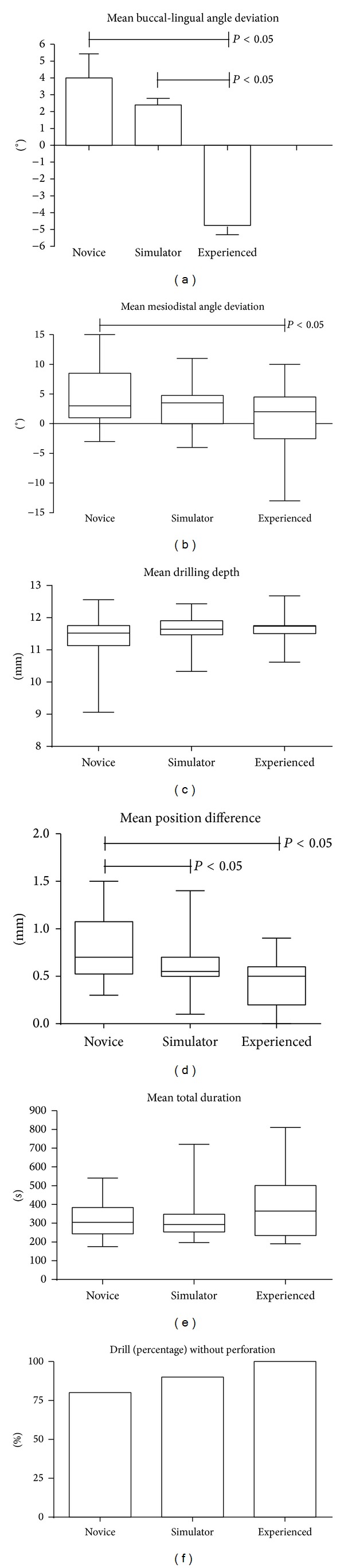
Comparative drilling outcomes of “Experienced,” “Simulation,” and “Novice” group on resin model. (a) Mean buccolingual angle deviation. (b) Mean mesiodistal angle deviation. (c) Mean drilling depth. (d) Mean position difference. (e) Mean total duration. (f) Site preparation (percentage) without perforation. Results are expressed as m ± SEM, *n* = 20 per group. *P < 0.05 versus Experienced.

Figure 4 shows the different parameters to highlight the quality of drilling observed in the three groups.

Regarding the difference in buccolingual angulation, there was a marked difference in operational approaches (Figure 4(a)). For the “Novice” group, the error outlined a drilling axis which tended to go from the buccal side to the lingual side with an average deviation angle of +4.0° ± 1.4° compared to the reference axis. With “Experienced” practitioners, the approach was reversed, the head of the implant facing the lingual side and the apical end oriented towards the buccal cortical; the average buccolingual deviation was −4.8 ± 1.1° (*t* = 5.004, *P* < 0.0001 versus “Novice”). After completing eight simulation sessions, 3rd year students in the “Simulator” group still drilled with a slightly buccolingually directed axis, the mean deviation being measured as an angle of +2.4 ± 0.7°. Although the significance level was not reached between the “Novice” and “Simulation” groups, the drilling direction was more accurate in the simulator group; however it was still far from the mean axis chosen by the “Experienced” group (*t* = 5.516, *P* < 0.0001, “Simulation" group versus “Experienced" group).

Less caricatural pattern was seen when measuring the difference in the mesiodistal angulation (Figure 4(b)). The experienced practitioners tended to follow instructions more precisely; the mean deviation from the reference was a mere 0.94 ± 1.33°. The novices were prone to drilling with a mesiodistal incline compared to the reference axis. The mesiodistal deviation was 4.71 ± 1.22° and the difference with the experienced practitioners was statistically significant (*t* = 2.085, *P* = 0.045). Students trained on the simulator had a better score (2.70 ± 0.89°) compared to “Novice” although this difference was not significant due to high variability of the values recorded in the “Novice” group (*t* = 1.350, *P* = 0.1856).

Another important factor is the drilling depth. It is often necessary to drill slightly deeper while avoiding the environing anatomical elements, especially in a situation where the dental nerve canal is near.  Figure 4(c) shows that all participants tended to make preparation by drilling slightly over the requested 11 mm. Values were 11.65 ± 0.08 mm in the “Experienced” group, 11.64 ± 0.11 in the “Simulation” group, and 11.43 ± 0.17 in the “Novice” one. Again, there was significant variability in the results from the “Novice” groups.

The positioning of the point of impact (centerline) on the ridge was another important factor of implant outcomes. When using the centering error parameter (Figure 4(d)), computed as the sum of the mean differences of position (mesiodistal and buccolingual directions) between each test and the virtual reference drilling, our analyses found that the results from “Experienced” were close to the expected ideal centering. Their error was only 0.43 ± 0.05 mm from the center. The centering error of “Novice” was more pronounced with a distance of 0.85 ± 0.09 mm from the ideal center (*P* < 0.001 versus “Experienced”). On the other hand, the “Simulator” group performed fairly well with a score of 0.57 ± 0.06 mm, a significant improvement compared to the “Novice” group (*t* = 2.502, *P* = 0.0168) but without reaching the level of the “Experienced” group.

While the average drilling time was about the same for all three groups (approximately 350 seconds, Figure 4(e)), the quality of the preparation of the implant site, estimated by the presence or absence of cortical perforation, was significantly different in the “Novice” group and the “Simulation” group when compared to the “Experienced” practitioners (Fisher test, *P* = 0.02). The frequency of perforation was 20% for “Novice” and 10% for the simulation trained students (Figure 4(f)).

## 4. Discussion

Implant surgery training, like all surgical training, is based on acquisition of fundamental knowledge while learning to perform procedures both efficiently and safely. To achieve a successful esthetic result, the very sensitive procedure of implant placement allows little room for error and demands a thorough, careful treatment planning combined with excellent clinical skills technique. Recently, it has been stressed to promote new learning environment evolving towards systems that can address the training needs of a growing number of practitioners with fewer instructors and yet allow numerous repetitions of specific procedures while objectively assessing the progress in acquiring skills, and all of this safely. In this particular context, we have studied benefits provided by a new haptic simulator.

Suebnukarn et al. [11] concluded that the use of haptic technology increased student performance in achieving basic dentistry procedures. This was already mentioned in a previous study by Buchanan [12], which showed that students' learning curve was significantly enhanced when they trained with the new technology. Sternberg et al. [13] demonstrated the usefulness of a haptic stimulator named Voxel-man towards learning apicectomy. From what we observed during implant site preparation, this technology helps students progress better and faster. The Prague Consensus Conference in 2008 was the first basis in an attempt to harmonize dental implant training in Europe [8]. This education must cover all fields from diagnosis to prosthetic treatment, including communication with the patient and legal aspects relative to implantology. If educators strive to make their teaching accessible, it is nonetheless necessary to develop methods to quickly and easily assess the progress that is made. The manual skills and gestures are surely more difficult to evaluate objectively than fundamental knowledge. Practical training on models or anatomical body parts involves heavy investment in equipment and material that must be renewed at each use. This was the approach we used when comparing drilling skills between novice students, stimulator trained students, and experienced practitioners. Digital simulators that avoid some of these expenses have been gaining attention since the early 2000s [14, 15]. Simulators with force feedback haptic arms have been successfully tested in restorative dentistry, oral surgery, and many medical fields [1–3, 11, 16–22] and in periodontology.

This guided us in selecting the type of simulator that we used to train the students in the “Simulation” group. A rapid progression was observable for the different parameters. The group having trained on the simulator perforated the external cortical bone twice less frequently than the “Novice” group that had only received lectures on surgery. Simulators should enhance the learning curve [12, 23] and provide tactile sensations that cannot be acquired during lectures. During this evaluation, the differences between experienced practitioners and novices were very obvious especially for parameters of assessment angulation (buccolingual and mesiodistal), centering the implant and perforation. Regarding the angular indication, the mesiodistal approach (front to back), definitely easier to appreciate, showed a steady progress towards a perfect angle. Large heterogeneity in the obtained performance was the general characteristics of the “Novice” group. The simulation allowed reducing the gap between students and suggesting that the proposed training exercises produced a favorable impact in the implementation of this act. The appreciation of the buccolingual angulation was less obvious at first glance. While we expected to see a difference in buccolingual angle close to 0° in “Experienced,” they chose mainly to position the implant with an angle from inside to outside and from above down with the tip of the implant led to the vestibular side. The other two groups have carried out the reverse. In fact, it appears that the clinical experience of “Experienced” has made them prefer a position angle allowing them to avoid puncturing the much finer lingual cortex. The appreciation of the depth was another important point. In our study, if the average depth of drilling did not differ significantly, the expertise and training with simulator reduced interindividual variability. Reproducibility is crucial for procedure certification and patient safety. In this respect, the work of Ioannou et al. [24, 25] based on parameters characterizing the drilling carried out by students and experienced practitioners is revealing. For these authors, a good command of the applied force allows for optimum drilling and faster performance by experienced practitioners rather than novices. In the present study, although we were not able to study the applied force, the small difference between the experienced practitioners for the drilling depth parameter was consistent with the results of Ioannou et al. [24, 25].

Procedural safety is an important issue [26]. Although virtual procedures cannot totally replace reality, haptic simulation and repeat training enables residents to be more confident during their first operation. Time is another important factor. Simulators should reduce the duration of procedures performed by undergraduate students or residents which is beneficial to both patient and practitioner [27]. In the present study the time spent on the drilling procedure diminished significantly with the simulator training. Other researchers [24, 25] have demonstrated that experienced practitioners need less time to prepare an implant site. In order to determine consistent results and to define training goals for different educational levels (undergraduate, graduate, and continuing education) it will be necessary to accomplish studies over a longer period of time including more exercises. This will enable educators to establish guide lines for using haptic technology towards preparing students for surgery [12, 23, 28].

Another important aspect is the use of surgical guides. During training the presence or absence of these guides considerably conditions the results. We observed, for instance, good progression in adopting the right drilling angle as long as the angulation guide was in place and a stagnation or slight regression once it was removed. However, during surgery, the operator will not have this sort of indication, so it is necessary to determine how long trainees are going to depend on the guides before progressively doing away with them. As is already observed in the practice of aviation and recently in medical education, the briefing-debriefing procedure plays a crucial role when using simulation for learning purposes [29]. Debriefing serves as a feedback in order to determine the need to correct some of the information gleaned through the learning action. At the same time, it enhances the reflection phase of the learning cycle [29].

## 5. Conclusion

This is an experimental approach to using a simulator for implant surgery training. The large number of practitioners to train, the increasing demand for safe clinical procedures, and the need for self-evaluation and self-training [28] are among the numerous reasons haptic simulation is drawing greater attention as a new and modern way to learn. Our present study has evidenced that 3rd year students trained with a simulator perform much better than students without prior training and that their performances soon approach those of more experienced practitioners. Haptic technology has a place in under graduate and graduate education as well as continuing education [30].

This new technology could, at least partially, overcome educational difficulties related to an increasing number of students to train with ever constant resources [31, 32]. To do so, software must be developed to simulate multiple and increasingly difficult situations thus to fulfill different pedagogic objectives for diverse universities and training centers.

## References

[1] Gallagher AG, Lederman AB, McGlade K, Satava RM, Smith CD (2004). Discriminative validity of the minimally-invasive surgical trainer in virtual reality (MIST-VR) using criteria levels based on expert performance. *Surgical Endoscopy*.

[2] Gallagher AG, Richie K, McClure N, McGuigan J (2001). Objective psychomotor skills assessment of experienced, junior, and novice laparoscopists with virtual reality. *World Journal of Surgery*.

[3] Gallagher K, Stephenson J, Brown RW, Holmes C, Ballester P (2005). Exploiting 3D spatial sampling in inverse modeling of thermochronological data. *Reviews in Mineralogy and Geochemistry*.

[4] Okuda Y, Bryson EO, DeMaria S (2009). The utility of simulation in medical education: what is the evidence?. *Mount Sinai Journal of Medicine*.

[5] Bilotta FF, Werner SM, Bergese SD, Rosa G (2013). Impact and implementation of simulation-based training for safety. *Scientific World Journal*.

[6] Perrenot C, Perez M, Tran N (2012). The virtual reality simulator dV-trainer is a valid assessment tool for robotic surgical skills. *Surgical Endoscopy*.

[7] Karle H, The Executive Council, World Federation for Medical Education (2008). International recognition of basic medical education programmes. *Medical Education*.

[8] Mattheos N, Albrektsson T, Buser D (2009). Teaching and assessment of implant dentistry in undergraduate and postgraduate education: a European consensus. *European Journal of Dental Education*.

[9] Lekholm U, Zarb G, Brånemark PI, Zarb GA, Albrektsson T (1985). Patient selection and preparation. *Tissue-Intergrated Prosthesess: Osseointegration in Clinical Dentistry*.

[10] Vázquez B, Ginebra MP, Gil FJ, Planell JA, López Bravo A, San Román J (1999). Radiopaque acrylic cements prepared with a new acrylic derivative of iodo-quinoline. *Biomaterials*.

[11] Suebnukarn S, Haddawy P, Rhienmora P, Jittimanee P, Viratket P (2010). Augmented kinematic feedback from haptic virtual reality for dental skill acquisition. *Journal of Dental Education*.

[12] Buchanan JA (2004). Experience with virtual reality-based technology in teaching restorative dental procedures. *Journal of Dental Education*.

[13] von Sternberg N, Bartsch MS, Petersik A (2007). Learning by doing virtually. *International Journal of Oral and Maxillofacial Surgery*.

[14] Luciano C, Banerjee P, DeFenti T (2009). Haptics-based virtual reality periodontal training simulator. *Virtual Reality*.

[15] Marras I, Nikolaidis N, Mikrogeorgis G, Lyroudia K, Pitas I (2008). A virtual system for cavity preparation in endodontics. *Journal of Dental Education*.

[16] Wierinck ER, Puttemans V, Swinnen SP, van Steenberghe D (2007). Expert performance on a virtual reality simulation system. *Journal of Dental Education*.

[17] Wierinck E, Puttemans V, Swinnen S, van Steenberghe D (2005). Effect of augmented visual feedback from a virtual reality simulation system on manual dexterity training. *European Journal of Dental Education*.

[18] van der Meijden OAJ, Schijven MP (2009). The value of haptic feedback in conventional and robot-assisted minimal invasive surgery and virtual reality training: a current review. *Surgical Endoscopy*.

[19] Quinn F, Keogh P, McDonald A, Hussey D (2003). A study comparing the effectiveness of conventional training and virtual reality simulation in the skills acquisition of junior dental students. *European Journal of Dental Education*.

[20] Moorthy K, Munz Y, Sarker SK, Darzi A (2003). Objective assessment of technical skills in surgery. *British Medical Journal*.

[21] Pohlenz P, Gröbe A, Petersik A (2010). Virtual dental surgery as a new educational tool in dental school. *Journal of Cranio-Maxillofacial Surgery*.

[22] Suebnukarn S, Hataidechadusadee R, Suwannasri N, Suprasert N, Rhienmora P, Haddawy P (2011). Access cavity preparation training using haptic virtual reality and microcomputed tomography tooth models. *International Endodontic Journal*.

[23] Buchanan JA (2001). Use of simulation technology in dental education. *Journal of Dental Education*.

[24] Ioannou I, Stern L, Kazmierczak E, Smith AC, Wise LZ (2010). Towards defining dental drilling competence. Part 2: a study of cues and factors in bone drilling. *Journal of Dental Education*.

[25] Ioannou I, Kazmierczak E, Stern L, Smith AC, Wise LZ, Field B (2010). Towards defining dental drilling competence. Part 1: a study of bone drilling technique. *Journal of Dental Education*.

[26] Abraham J, Wade DM, O’Connell KA, Desharnais S, Jacoby R (2011). The use of simulation training in teaching health care quality and safety: an annotated bibliography. *The American Journal of Medical Quality*.

[27] Haque S, Srinivasan S (2006). A meta-analysis of the training effectiveness of virtual reality surgical simulators. *IEEE Transactions on Information Technology in Biomedicine*.

[28] Mattheos N, Ucer C, van de Velde T, Nattestad A (2009). Assessment of knowledge and competencies related to implant dentistry in undergraduate and postgraduate university education. *European Journal of Dental Education*.

[29] Fanning RM, Gaba DM (2007). The role of debriefing in simulation-based learning. *Simulation in Healthcare*.

[30] McGaghie WC, Issenberg SB, Petrusa ER, Scalese RJ (2010). A critical review of simulation-based medical education research: 2003–2009. *Medical Education*.

[31] Cederberg RA, Bentley DA, Halpin R, Valenza JA (2012). Use of virtual patients in dental education: a survey of U.S. and Canadian dental schools. *Journal of Dental Education*.

[32] Morton J, Cumming A, Cameron H (2007). Performance-based assessment in undergraduate medical education. *Clinical Teacher*.

